# Posture dependent factors influence movement variability when reaching to nearby virtual objects

**DOI:** 10.3389/fnins.2022.971382

**Published:** 2022-10-25

**Authors:** Preyaporn Phataraphruk, Qasim Rahman, Kishor Lakshminarayanan, Mitchell Fruchtman, Christopher A. Buneo

**Affiliations:** Visuomotor Learning Laboratory, School of Biological and Health Systems Engineering, Arizona State University, Tempe, AZ, United States

**Keywords:** vision, proprioception, noise, planning, execution

## Abstract

Reaching movements are subject to noise arising during the sensing, planning and execution phases of movement production, which contributes to movement variability. When vision of the moving hand is available, reach endpoint variability appears to be strongly influenced by internal noise associated with the specification and/or online updating of movement plans in visual coordinates. In contrast, without hand vision, endpoint variability appears more dependent upon movement direction, suggesting a greater influence of execution noise. Given that execution noise acts in part at the muscular level, we hypothesized that reaching variability should depend not only on movement direction but initial arm posture as well. Moreover, given that the effects of execution noise are more apparent when hand vision is unavailable, we reasoned that postural effects would be more evident when visual feedback was withheld. To test these hypotheses, participants planned memory-guided reaching movements to three frontal plane targets using one of two initial arm postures (“adducted” or “abducted”), attained by rotating the arm about the shoulder-hand axis. In this way, variability was examined for two sets of movements that were largely identical in endpoint coordinates but different in joint/muscle-based coordinates. We found that patterns of reaching variability differed in several respects when movements were initiated with different arm postures. These postural effects were evident shortly after movement onset, near the midpoints of the movements, and again at the endpoints. At the endpoints, posture dependent effects interacted with effects of visual feedback to determine some aspects of variability. These results suggest that posture dependent execution noise interacts with feedback control mechanisms and biomechanical factors to determine patterns of reach endpoint variability in 3D space.

## Introduction

Variability is inherent in movement production and studies of movement variability have and continue to inform our understanding of coordinate transformations, motor learning, and optimal motor control (Gordon et al., 1994; Mcintyre et al., 1997, 1998; Harris and Wolpert, 1998; Van Beers, 2009). Movement variability has been attributed in part to “neural noise” arising during the encoding and integration of sensory signals and/or the planning and generation of motor commands (Faisal et al., 2008). Until fairly recently conventional wisdom has held that noise is detrimental to motor behavior (Harris and Wolpert, 1998 Herzfeld and Shadmehr, 2014). However, recent work has shown that a component of movement variability appears to arise from a gradual accumulation of the random effects of planning noise, a phenomenon which could benefit motor learning by fostering exploration in the motor command space (Van Beers et al., 2013). Similarly, the observation that baseline levels of movement variability can predict the rate at which individual human participants learn motor tasks suggests that some component of neural noise might actually be advantageous or even necessary for motor learning to occur (Wu et al., 2014). Recent work demonstrating that a covariation of slow drifts in neural and behavioral variability is well explained by a simple model of error-corrective learning, appears to provide additional support for this idea (Chaisanguanthum et al., 2014).

For reaching movements, noise can arise during the initial encoding and/or updating of the hand and/or goal location (“sensory noise”), during the transformation of sensory signals into motor commands (“planning noise”) or during the transformation of commands into movement (“execution noise”) (Buneo et al., 1995; Van Beers et al., 2004; Osborne et al., 2005; Churchland et al., 2006a,b; Shi and Buneo, 2012). As a result, the effects of noise on reaching variability are highly context dependent. For example, when the hand is visible during movement, variability tends to be greater in depth than along other axes, reflecting uncertainty associated with visual localization of the hand and/or targets in depth (Mcintyre et al., 1997, 1998; Carrozzo et al., 1999; Apker and Buneo, 2012). In contrast, *without* visual feedback of the moving hand variability is greatest along an axis that is collinear with the direction of movement (Gordon et al., 1994; Mcintyre et al., 1997, 1998). These latter effects do not appear to be related to planning noise but noise associated with execution, particularly during the terminal phases of movement (Van Beers et al., 2004 Apker and Buneo, 2012).

Given that execution noise acts in part at the muscular level (Faisal et al., 2008), it is reasonable to assume that reaching variability should depend not only on movement direction but initial arm posture as well. However, effects of arm posture on movement variability have not to date been extensively characterized experimentally. In a previous study of three-dimensional (3D) memory-guided reaching movements performed with diminished or absent visual feedback, movement endpoint distributions varied in orientation between starting positions as well as with the hand used to make the movement, suggesting a dependence on arm posture (Mcintyre et al., 1998). Similarly, in a simulation study of 2D planar reaching movements performed without feedback, variability in initial movement directions resulting from both planning and execution noise were shown to rotate systematically with changes in initial hand position/arm posture, maintaining a roughly fixed relationship with respect to the arm rather than remaining fixed in space (Shi and Buneo, 2012). These studies support the idea that variability should depend upon both movement direction and arm posture, at least in the absence of visual feedback and when changes in arm posture are largely coplanar with planned movement directions (as is the case of 2D planar movements).

For movements performed in 3D space, changes in initial hand position rarely involve changes in arm posture that are entirely coplanar with planned movement directions and can in fact involve postural changes that are orthogonal to these directions, as when rotating the arm about an axis connecting the shoulder to the hand. Postural changes that are orthogonal to planned movement directions are essentially irrelevant to the planning of movement vectors, as they don’t change the position of the hand relative to the goal location, but are still highly relevant to the planning of dynamics and execution due to their effects on muscle mechanical actions and joint torques (Buneo et al., 1995, 1997; Soechting et al., 1995; Sober and Sabes, 2003). However, the effects of such postural changes on movement variability are difficult to predict *a priori*. As a result, in this study we quantified the movement variability associated with memory-guided reaching movements initiated from a single starting position to three targets contained in a frontal plane. At the starting position, participants matched one of two desired arm configurations (“adducted” and “abducted”) by rotating the arm about the shoulder-hand axis, thereby maintaining a constant endpoint position. In this way, changes in arm posture were orthogonal to planned movement directions. In addition, movements were performed with and without vision of the moving hand. Based on previous studies we hypothesized that the orientation of movement endpoint distributions would vary with both target direction and arm posture and that these variations would be more evident when vision of the moving hand was withheld.

## Materials and methods

### Participants

Ten participants (5 men and 5 women) between the ages of 19 and 53 were recruited to perform the experiment. Participants were briefed on the experimental procedure, which involved reaching with the right arm to targets in 3D space using the same starting fingertip position but different initial arm postures, but were naïve to the actual purpose of the experiment. The protocol was approved by the Arizona State University Institutional Review Board (IRB) and participants read and signed an IRB approved informed consent form before participating in the experiment, which was conducted in accordance with the principles expressed in the Declaration of Helsinki. Handedness was assessed using the Edinburgh Handedness Inventory (short form). All but one of the participants were determined to be right-handed.

### Apparatus and data acquisition

The experimental apparatus consisted of a large, standing metal frame that supported a 3D stereoscopic monitor (Dimension Technologies, Rochester). The monitor projected images through an opening in the metal frame onto a reflective mirror embedded in a metal shield. The metal shield was oriented at a 45^°^ angle with respect to the monitor and enabled the participants to see the projected images on the mirror. The metal shield also served to block the participants’ arms from view. Participants positioned their heads on a chin rest which aligned their eyes with the center of the mirror and were asked not to look away from the mirror during the entire experiment. Participants were also asked to limit repositioning of their body during the experiment.

An active-LED-based motion tracking system was used to track movements of the arm (Visualeyez VZ-3000 motion tracker; Phoenix Technologies, Burnaby, British Columbia; 250-Hz sampling rate; 0.5-mm spatial resolution). Three (3) LEDs were placed on each participants’ fingertip, elbow, and shoulder, respectively. The position of the fingertip LED was fed back to the participant in near real-time within a virtual reality environment developed in Vizard (WorldViz, Santa Barbara, CA). The fingertip position, starting position and targets were displayed as green spheres of ∼5 cm diameter in the VR environment. To aid in depth perception, a cube object was also rendered in the VR environment. Monitoring of fingertip position and arm configuration, as well as interfacing with the VR environment was accomplished via a custom program developed in LabVIEW (National Instruments Corporation, Austin, Texas) which also downsampled the fingertip position at 125 Hz.

### Experimental design

Participants were required to make memory-guided reaching movements to three targets using one of two initial arm configurations and with or without visual feedback of the fingertip. We used a memory-guided task to be consistent with previous studies of reaching in three dimensions (e.g., Mcintyre et al., 1998). The starting position of the hand was located on the body midline at approximately shoulder level and all targets were located 11.7 cm from the starting position. One target was located directly ahead of the starting position on the body midline (target M) at an elevation angle of 60^°^ with respect to the horizontal plane containing the starting position. The other two targets were located 45^°^ to the left and right of the starting position (targets L and R, respectively) and at elevation angles of 45^°^. Targets L and R appeared at approximately eye level, while target M appeared slightly superior to the lateral targets. Given the arrangement of the targets, on a given trial participants were required to reach upward and in depth from the starting position and either directly forward (0^°^) or slightly leftward (−45^°^) or slightly rightward (45^°^).

Trials began with the illumination of the starting position. Once participants acquired the starting position with their fingertip and maintained that position for 1,000 ms the start position was extinguished and a target was illuminated for 300 ms, which was then also extinguished. Participants were then required to withhold making a movement to the target during an ensuing memory period of 500–1,500 ms. At the end of the delay period a 60 Hz tone was generated, which served as the “go” cue to begin the movement. If participants completed their movements within 1,000 ms and maintained their fingertip at the perceived target location for 1,250 ms, another auditory tone was generated, indicating the end of the trial. Vision of the fingertip was available to the participant throughout the trial in the vision (V) condition but was removed at the go cue in the non-vision (NV) condition. Feedback condition (V, NV) and target direction were randomly varied on trial-by-trial basis.

**FIGURE 1:**
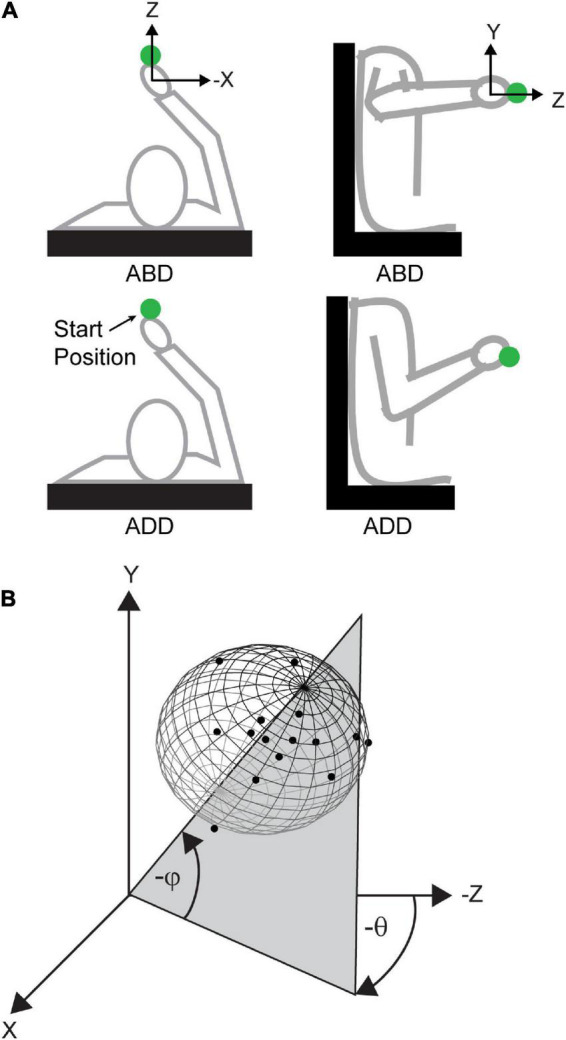
Arm postures and angles used to define variability ellipsoid orientations. **(A)** Top-down (left) and lateral (right) views of the abducted (ABD) and adducted (ADD) postures. **(B)** Simulated 3D endpoint distribution showing definitions for azimuth (θ) and elevation (φ). Azimuth was defined as the angle in the x-z plane and was positive going from the negative *z*-axis toward the negative *x*-axis. Elevation was defined as the angle from the x-z plane and was positive going toward the negative *y*-axis.

Trials were organized into two blocks, with each block employing either an “adducted” or “abducted” arm posture at the starting position (Figure 1A). Arm posture was changed by rotating the arm about the shoulder-hand axis. In this way, the same starting position was maintained, thereby ensuring that planned movement vectors were largely identical between postures. The order of the blocks were randomized across participants. The angle that the arm plane (i.e., the plane containing the upper arm and forearm) made with horizontal was used to define arm posture. An arm plane angle of 0^°^corresponded to full abduction and 90^°^ indicated full adduction. For the abducted block, participants were required to maintain their posture between 0 and 45^°^ and for the adducted block, a posture between 45 and 90^°^ was required. If at any point during the trial participants failed to maintain their posture within the required range, a 1 kHz tone was generated, cueing them to reposition. A total of 90 trials were performed in each block (15 V trials and 15 NV trials to each of three targets). Participants were given an approximately 1 min rest period every 15 trials within a block as well as between blocks to minimize fatigue due to elevating the limb for extended periods.

Participants had no knowledge of the trial parameters and were given instructions to move quickly and accurately to the targets using a single uncorrected movement. A trial was considered successful if the participant maintained the initial starting position and arm posture, reached a target within the required spatial and temporal windows, and maintained position at the end of the movement for 1,200 ms. If any of the criteria for a successful trial were not met, the trial was aborted and repeated later in the block.

### Data analysis

Movement data were smoothed using digital low-pass filters (4th order, 6 Hz cutoff). The beginning and end of each movement was defined as the points at which the tangential movement velocity exceeded or fell to 10% of its peak value. Data from trials where the tangential velocity exhibited multiple peaks or other irregularities were discarded (2% of all trials).

Movement endpoints were then sorted according to target direction, visual feedback condition, and arm posture to form 3D movement endpoint distributions. Although our primary focus was on the orientation of these distributions we also analyzed their sizes (volumes) and shapes (aspect ratios). To determine volume and aspect ratio we first calculated a 95% tolerance ellipsoid for each endpoint distribution (Khachiyan and Todd, 1993; Khachiyan, 1996; Moshtagh, 2009). The volume of each ellipsoid was then quantified as follows:


$$V=\frac{4\,\pi }{3}\,x\,y\,z$$

where *x* represents the radius of the major axis of the ellipsoid and *y* and *z* refer to the radii of the minor axes. We calculated the aspect ratio of each ellipsoid as the ratio of the radius of the major axis to that of the smaller of the radii of the minor axes. Volumes and aspect ratios greater than 3 scaled median absolute deviation (MAD) away from the median were considered outliers and were excluded from subsequent analyses (∼7% of ellipsoids).

To quantify the orientation of each endpoint distribution, principal components analysis (PCA) was used (Mcintyre et al., 1997, 1998; Carrozzo et al., 1999; Apker et al., 2010; Apker and Buneo, 2012). Here, the first eigenvector derived from PCA was used as an indicator of the principal axis of movement variability and the orientation of this axis was parameterized by its azimuth [angle within the x-z plane (θ)] and elevation [angle out of the x-z plane (φ)] (Figure 1B). First eigenvectors that accounted for greater than half of the total variance (∼90 of the distributions) were included in subsequent analyses.

In addition to analyses conducted on movement endpoints, some analyses were also conducted at earlier points along the movement trajectories. Specifically, we quantified differences in ellipsoid azimuth (Δθ) between initial arm postures as a function of movement extent for each visual condition. Angular means and standard errors were calculated at 10–100% of the total movement extent, in 5% increments.

#### Statistical analyses

Statistical analyses were conducted separately for each target (L, M, and R) and were implemented in SPSS version 27 (IBM Corp.). Effects of visual condition (V, NV) and initial arm posture (ADD, ABD) on kinematic (peak velocity, path length) and behavioral parameters (reaction and movement times) were analyzed using two-factor repeated measures analyses of variance (ANOVA). Since the goal of these analyses was to determine if initial arm posture and visual condition had effects on kinematics and behavior as a whole, the set of four tests conducted within each target were treated as a “family.” Therefore, to control for family-wise error rate, a Bonferroni corrected alpha of 0.0125 was used for these tests. Effects of visual condition and initial arm posture (ADD, ABD) on final ellipsoid volumes, aspect ratios, and orientations (azimuth, elevation) were also analyzed using two-factor ANOVAs. Here, the two orientation parameters (azimuth, elevation) were treated as belonging to the same family and an alpha of 0.025 was used for these analyses. For all other analyses, an alpha of 0.05 was used. Note that in Table 1 and Supplementary Table 2, *p*-values have been adjusted in accordance with the above procedures to allow for comparison against a fixed alpha of 0.05.

**TABLE 1:** Two factor repeated measures ANOVA results for the effects of initial arm and visual condition on ellipsoid volumes, aspect ratios, and orientations (azimuth, elevation).

		L			M			R		
				
		df	*F*	*p*	df	*F*	*p*	df	*F*	*p*
Volume	Vision	**(1, 6)**	**22.941**	**0.003**	**(1, 7)**	**18.951**	**0.003**	**(1, 8)**	**31.941**	**<0.001**
	Posture	(1, 6)	<0.001	0.985	(1, 7)	0.291	0.606	(1, 8)	0.235	0.641
	Interaction	**(1, 6)**	**8.085**	**0.029**	(1, 7)	0.614	0.459	(1, 8)	0.118	0.740
Aspect ratio	Vision	(1, 6)	2.781	0.146	**(1, 5)**	**15.451**	**0.011**	(1, 5)	0.052	0.828
	Posture	(1, 6)	0.107	0.754	(1, 5)	3.668	0.114	(1, 5)	3.604	0.116
	Interaction	**(1, 6)**	**8.046**	**0.030**	(1, 5)	0.025	0.879	(1, 5)	0.015	0.908
Azimuth (θ)	Vision	(1, 8)	0.081	1	(1, 6)	0.331	1	(1, 3)	0.200	1
	Posture	(1, 8)	0.525	0.978	**(1, 6)**	**9.231**	**0.046**	(1, 3)	0.263	1
	Interaction	(1, 8)	0.357	1	(1, 6)	0.335	1	(1, 3)	1.013	0.776
Elevation (φ)	Vision	(1, 8)	1.499	0.512	(1, 6)	0.660	0.896	(1, 3)	0.250	1
	Posture	(1, 8)	0.167	1	(1, 6)	0.686	0.878	(1, 3)	1.789	0.546
	Interaction	(1, 8)	0.209	1	(1, 6)	0.356	1	(1, 3)	0.006	1

*P*-values for azimuth and elevation have been Bonferroni corrected as described in section “Materials and methods”. Cells in bold indicate statistically significant effects at an alpha level of 0.05.

Given that our orientation data were angular in nature, each angle was also analyzed using a two-factor circular ANOVA, implemented in Matlab, 2019 (The Mathworks, Inc.), using the CircStat toolbox (Berens, 2009). However, since the overall results were very similar to those reported for the linear analyses, the results of these circular analyses are not reported here.

### Results

As expected, initial arm orientations differed between the two instructed arm postures but were consistent between visual conditions and across movement directions (Supplementary Table 1). For the ADD posture, average arm orientations (rounded to the nearest degree) were approximately 60^°^ for all movement directions in both conditions. In contrast, average arm orientations for the ABD posture were approximately 25^°^ for all directions in both conditions. To assess the extent to which this ∼35^°^ difference in initial arm posture affected the overall performance of the participants in this experiment, we computed several standard behavioral and kinematic performance measures (Supplementary Table 1) and analyzed their dependence on the visual conditions and initial arm posture using two-factor, repeated measures ANOVAs (Supplementary Table 2). We found that there was a significant main effect of arm posture on peak tangential velocity for movements to the middle target, with velocities for the ADD posture tending to be greater than those for the ABD posture. This finding has implications for understanding the sources of posture dependent differences in movement variability, as discussed below.

**FIGURE 2:**
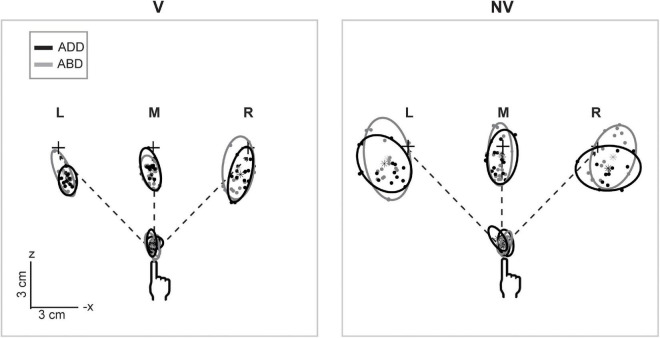
Top-down view of movement starting points and endpoints and associated 95% confidence ellipsoids for reaches to the left (L), middle (M), and right (R) targets for a representative participant. Dashed lines show the straight-line paths to each target. Ellipsoids were generally larger and more variable in shape and orientation in the NV condition. Effects of arm posture were also more apparent in the NV condition, particularly with regard to ellipsoid positions and orientations.

Figure 2 shows horizontal plane views of the movement endpoints and variability ellipsoids for a single participant in each experimental condition. In the V condition, the spatial distributions of the endpoints were relatively compact, resulting in smaller ellipsoids. In contrast, endpoints in the NV condition were more dispersed, indicating that the absence of hand visual feedback led to greater overall variability. In addition to being more compact, ellipsoids in the V condition were also more anisotropic and more consistent in orientation for a given movement direction than those in the NV condition, consistent with previous studies employing both memory-guided and reaction time tasks (Mcintyre et al., 1997, 1998; Carrozzo et al., 1999; Apker et al., 2010; Apker and Buneo, 2012). For this participant, initial arm posture appeared to have negligible effects on ellipsoid size, shape, and orientation in the V condition. In the NV condition, however, changes in posture resulted in more noticeable differences in ellipsoid orientation, though these rotations were inconsistent in sign and magnitude across movement directions. Lastly, changes in initial arm posture were also associated with differences in average endpoint positions, as indicated by their shifted positions in space, which again were greater in the NV condition.

**FIGURE 3:**
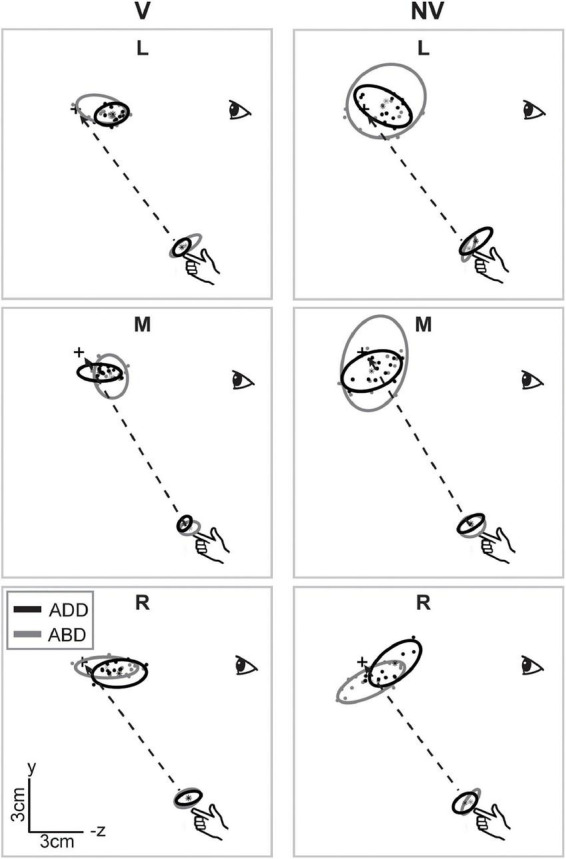
Lateral view of movement endpoints and confidence ellipsoids for reaches to each target for the participant shown in Figure 2. Dashed lines show the straight-line paths to each target. In the V condition, axes of maximum variability appeared to be better aligned with sight lines to the targets than with planned movement directions (dashed lines). In the NV condition, these axes were not consistently aligned with either planned movement directions or lines of sight. Ellipsoid size, shape, position, and orientation also appeared to vary more with arm posture in the NV condition, particularly for targets L and M.

Figure 3 shows a sagittal view of the movement endpoints and ellipsoids from the same participant. As in the horizontal plane, movement endpoint distributions were more compact and more consistent in orientation in the V condition compared to the NV condition. Interestingly, axes of maximum variability were not well aligned with planned hand movement directions (dashed lines). Instead, these axes were better aligned with the approximate lines of sight, suggesting variability was more strongly influenced by uncertainty in visually estimating the position of the hand and/or targets in depth than by execution related factors. In the NV condition, ellipsoids were larger and more variable in shape and orientation. In addition, axes of maximum variability were not consistently aligned with either planned movement directions or lines of sight. As in the sagittal plane view, arm posture had more noticeable effects on orientation and average endpoint positions in the NV condition, but these effects again differed across directions.

**FIGURE 4:**
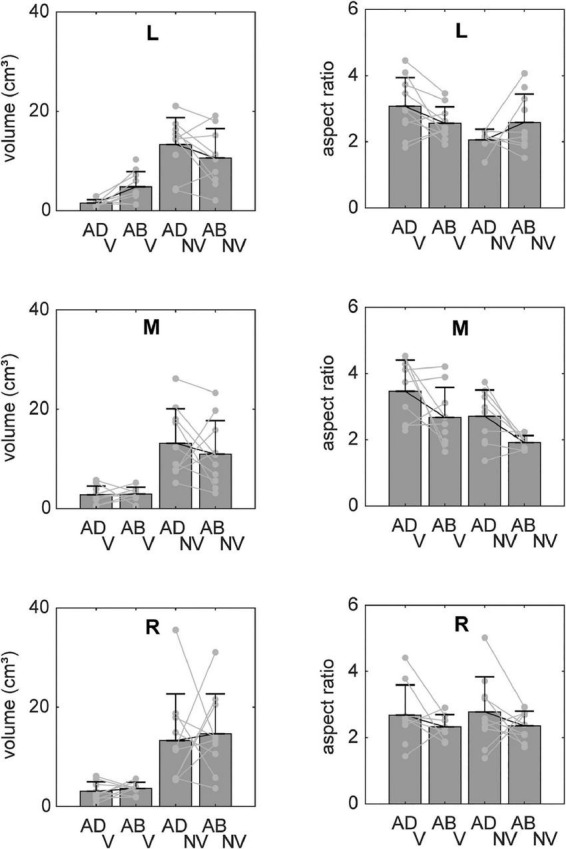
Average volumes and aspect ratios (±SD) for reaches to each target. Individual participant data (light gray lines) are superimposed on each set of bars. Volumes were generally much larger in the NV condition and also appeared to show a dependence on arm posture, though the nature of these postural effects varied between visual conditions and across targets. Aspect ratios showed less of a dependence on visual condition but also varied with initial arm posture in a manner that differed across targets and between visual conditions.

Systematic effects of vision and arm posture were more evident when data were combined across participants. Figure 4 shows bar plots of the ellipsoid volumes and aspect ratios for all experimental conditions. In the V condition (left column), average volumes were generally small and varied little across movement directions. In the NV case, volumes were much more variable and were typically more than twice as large, but still varied little across directions. Regarding effects of arm posture, for the left target, volumes were slightly larger for the ABD than for the ADD posture in the V condition, a trend which reversed for the NV condition. For the other directions, effects of posture were less clear, due in part to a lack of consistency in volume changes from participant to participant (light gray points and lines). Two-factor, repeated measures ANOVAs confirmed that there were statistically significant effects of vision for all targets, as well as a significant vision-posture interaction for the left target (Table 1). No other significant effects were found.

Vision and arm posture also had some effects on ellipsoid shapes, though similar to volume, these effects differed across movement directions. For the left target, aspect ratios were slightly larger (and therefore more elongated) for the ADD than for the ABD posture in the V condition, a trend which again reversed for the NV condition. For the middle target, aspect ratios in the V condition were somewhat larger than those in the NV condition and also appeared to be generally larger for the ADD posture. This apparent postural effect also appeared to be present for the right target, though to a lesser extent. Some but not all of these trends were confirmed by statistical analysis: for the left target there as a statistically significant vision-posture interaction, and for the middle target a significant effect of vision (Table 1). Otherwise, no statistically significant effects of vision, posture or their interaction on aspect ratio were found.

**FIGURE 5:**
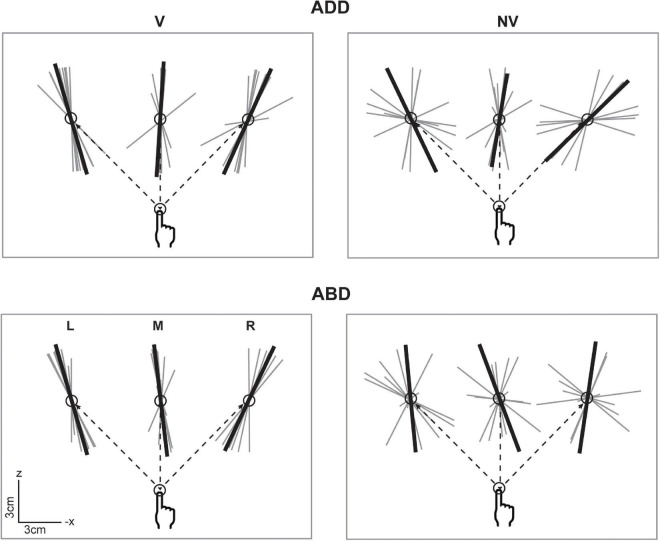
Top-down view of the individual (gray) and average (black) principal axes of variability in the V condition. Axes were scaled to an arbitrary length for visualization purposes. Dashed lines show the straight-line paths to each target. Average axes were more convergent toward the starting hand position in the V condition than in the NV condition. In the V condition, the average axis for the middle target demonstrated a small counter clockwise rotation as initial arm posture changed from ADD to ABD. A larger magnitude rotation in the same direction was also observed for this target in the NV condition, as well as for the right target.

Overall, the data in Figure 4 suggest that while vision had relatively strong effects on endpoint variability, particularly on ellipsoid volumes, postural effects were weaker and more idiosyncratic in nature. More consistent effects of arm posture were found with respect to ellipsoid orientation. Figure 5 shows horizontal plane views of the principal axes of variability for all participants in each condition, which allows visualization of the azimuthal component of orientation. In the V condition, individual axes (thin gray lines) were generally oriented closer to the average (thick black lines), i.e., they were less dispersed than those in the NV condition. In addition, looking across directions, average axes were more convergent toward the starting hand position (and therefore body midline) in the V condition than in the NV condition. In the V condition, orientations for the left and right target did not appear to vary between arm postures. For the middle target, however, a small counter clockwise rotation was observed when arm posture changed from ADD to ABD. A similar but larger rotation was also observed for this target in the NV condition, as well as for the right target. Thus, although visual feedback generally resulted in more spatially convergent axes of variability, arm posture changes appeared to have some consistent additional effects on the azimuthal orientation of these axes, primarily for the middle target.

**FIGURE 6:**
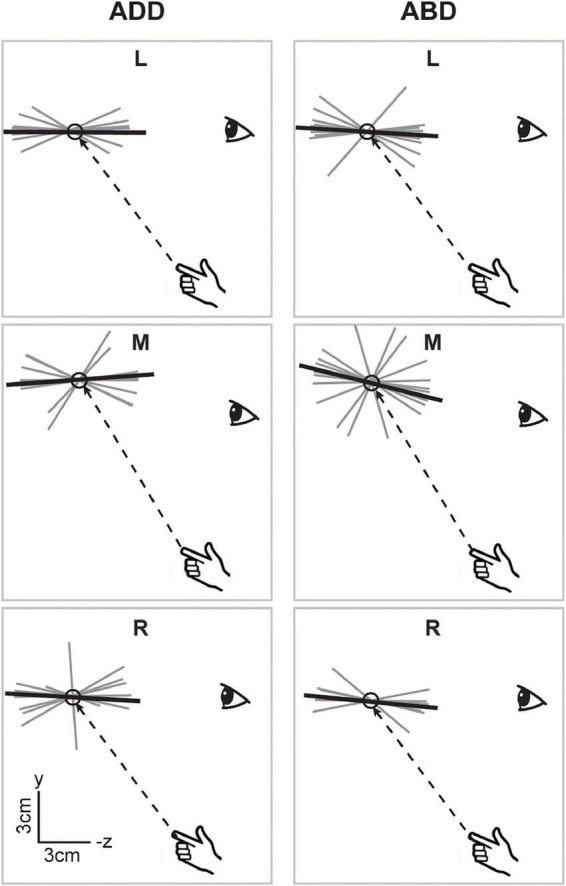
Lateral view of the individual (gray) and average (black) principal axes of variability in the V condition. Dashed lines show the straight-line paths to each target. Average axes were generally aligned along subjects’ approximated sight lines to the targets. Apparent effects of posture were only observed for the middle target and manifested as a downward rotation as posture changed from ADD to ABD.

**FIGURE 7:**
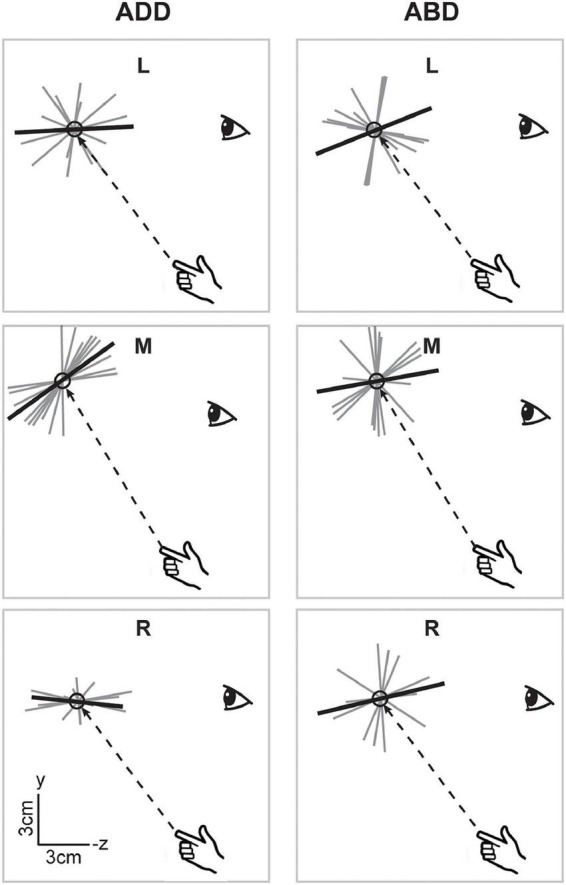
Lateral view of the individual and average principal axes of variability in the NV condition. Figure conventions follow those of Figure 6. In some cases (e.g., ADD L), average axes were roughly aligned with the approximated sight lines to the targets. More generally however axes were pitched upward, reminiscent of previous findings for memory-guided reaches (Mcintyre et al., 1997, 1998). Similar to the V condition, the axis for the middle target appeared to rotate downward as arm posture changed from ADD to ABD.

When viewed in the sagittal plane, axes for the V condition (Figure 6), showed little variation in elevation across directions or between arm postures. For the left and right targets, axes were aligned along participants’ approximated sight lines to the targets, as previously shown for the individual participant in Figure 3, regardless of initial arm posture. For the middle target, axes did appear to differ somewhat in orientation between postures, being pitched slightly upward for the ADD posture (when viewed from the participant’s perspective) but downward for the ABD posture. In the NV condition (Figure 7), individual axes were again more variable. For some directions (L and R for ADD) average axes were roughly aligned with the approximated sight lines to the targets. For the others, these axes were generally pitched upward, reminiscent of previous findings for memory-guided reaches (Mcintyre et al., 1997, 1998). Apparent effects of posture were also observed but were inconsistent across targets. However, for the middle target this effect was similar to what was observed in the V condition, i.e., a downward rotation as posture changed from ADD to ABD.

**FIGURE 8:**
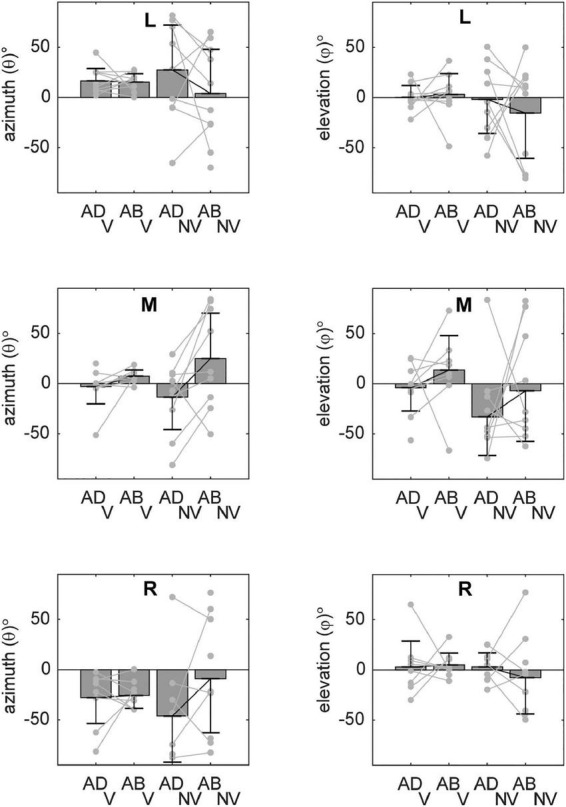
Average orientations of the principal axes of variability (±circular SDs) for each target. Individual participant data are superimposed on each set of bars (light gray lines). Azimuth angles (θ) grossly followed changes in required movement direction. Elevation angles (φ) were more variable but were close to 0^°^ in many cases, indicative of largely horizontal orientations. For the middle target (M), a change in arm posture from ADD to ABD was associated with increasing values of θ (particularly in the NV condition), consistent with a counterclockwise rotation in the horizontal plane (cf. Figure 5).

Although Figures 5–7 provided evidence that average orientations varied with the visual conditions and, to some extent, arm posture, it is difficult to appreciate from these plots how such changes manifested at the individual participant level. Figure 8 shows the ellipsoid orientation data for all participants superimposed on bar plots of the average orientations. In the V condition, azimuth angles (θ, left column) grossly followed changes in required movement direction, decreasing as movement direction varied from left to right. Elevation angles (φ, right column), though more variable, hovered around 0^°^, indicative of largely horizontal orientations. Arm posture appeared to have a small but consistent effect on θ (and possibly φ) for the middle target, increasing from ADD to ABD, but no other consistent effects were apparent for this condition. In the NV condition, variability was markedly increased relative to the V condition. However, for the middle target, θ again appeared to change with arm posture in a relatively consistent way across participants, increasing from ADD to ABD. An ANOVA confirmed that there was a statistically significant effect of arm posture on θ for the middle target (Table 1). Although larger changes in θ were observed in the NV condition (∼38^°^) compared to the V condition (∼11^°^) for this target, no statistically significant effect of the visual conditions nor a significant interaction between vision and posture were found.

**FIGURE 9:**
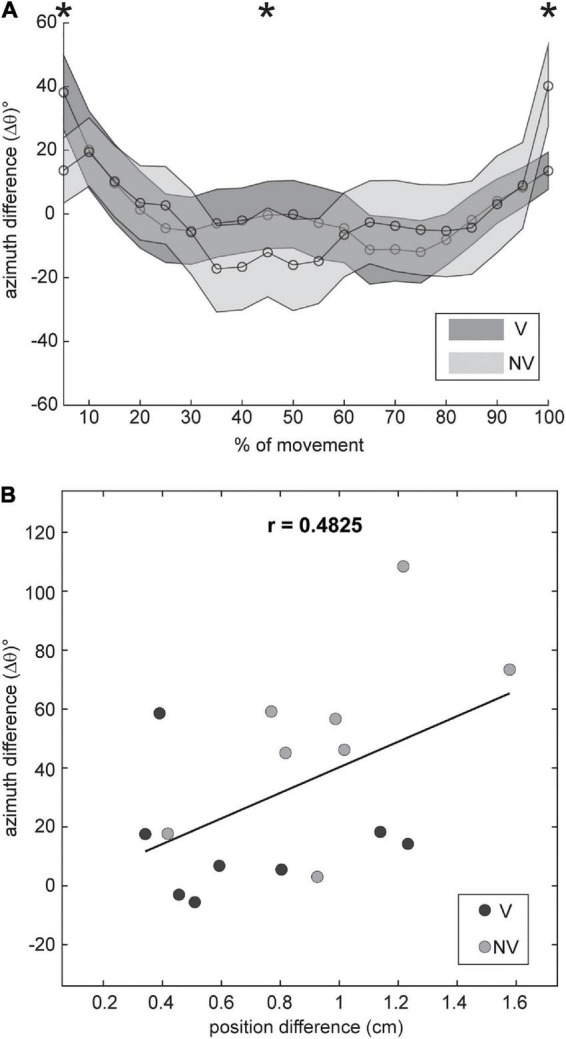
**(A)** Mean within-participant differences in azimuth between arm postures (±SE), plotted as a function of movement extent for both feedback conditions. Data were smoothed with a low pass filter prior to plotting. Statistically significant, posture dependent differences in azimuth (*) were evident very early (5%), near the midpoint of the movements (45%) and at the endpoint (100%). **(B)** Azimuth differences at the endpoint plotted against differences in mean endpoint position for each participant. Differences in azimuth were moderately correlated with differences in mean endpoint position.

Our analyses revealed that changes in initial arm posture did exert systematic effects on the orientation of movement endpoint distributions, but these effects were limited to changes in azimuth (θ) for movements to the middle target. Such effects could be attributed to at least two factors: posture dependent noise associated with movement planning (which should be evident from the very beginning of the movements up to the endpoint) and posture dependent execution noise (which should be more evident near the end of the movements). To gain insights into the relative contributions of these two factors we subtracted the azimuth values for the ADD posture from those for the ABD posture (Δθ) and plotted these differences as a function of movement extent (Figure 9A). If the effects of arm posture at the endpoint were due to planning noise, we reasoned that Δθ should be consistently non-zero throughout the entire extent of the movements. In contrast, if these effects were due to execution noise, then values of Δθ should be close to zero throughout most of the movements but increase near the endpoints. Figure 9A shows that Δθ was initially positive for both visual conditions shortly after movement onset (5–10%), close to zero (V condition) or slightly negative (NV condition) near the middle of the movements, and positive again at the movement endpoints (100%). Two-factor, repeated measures ANOVAs conducted at each percentage of movement extent confirmed these observations: statistically significant effects of arm posture were found only at 5% (*F* = 6.142; *p* = 0.042), 45% (*F* = 11.877; *p* = 0.007) and, as shown in Table 1, 100% of total movement extent. The finding that postural effects were not consistently present throughout the movements suggests that planning noise played only a marginal role in determining posture related orientation differences at the endpoint and that execution noise was likely a larger factor.

Biomechanical factors may have also contributed to posture related orientation differences at the endpoint. In these experiments, mean endpoint positions for a given target often differed between initial arm postures, as indicated by the shifted positions of the ellipses in Figures 2, 3, and these differences were also generally greater in the NV condition. This suggests that factors related to the control of final position might have influenced endpoint variability as well. To explore this possibility, we plotted differences in ellipsoid azimuth (Δθ) against corresponding differences in mean endpoint positions (“position difference”), using combined data from both visual conditions (Figure 9B). Overall, these differences were found to be moderately positively correlated (*r* = 0.48). Moreover, a linear regression analysis showed that differences in mean endpoint positions accounted for a substantial proportion of the variance in azimuth differences (*R*^2^ = 0.23), though the results of this regression analysis were not statistically significant (*F* = 4.248; *p* = 0.058). Thus, the extent to which position dependent biomechanical factors (e.g., limb mechanical impedance) played a role in determining patterns of endpoint variability in this experiment is unclear.

## Discussion

Previous work has shown that reach endpoint variability depends in part on internal noise arising during movement execution, particularly when vision of the moving hand is unavailable. Given that execution noise is thought to arise at least in part during muscle activation, we reasoned that variability arising from execution noise should depend not only on movement direction but initial arm posture as well. To test this hypothesis, we quantified patterns of variability that resulted when memory-guided reaching movements were executed in three directions using one of two initial arm configurations, which were attained by rotation about the shoulder-hand axis. In this way, effects of arm posture were examined for two sets of planned movement trajectories that were largely identical in endpoint coordinates but different in joint coordinates. In addition, movements were performed with and without vision of the moving hand (V and NV conditions, respectively). We found that patterns of reach endpoint variability differed in several respects for movements initiated with different arm postures, though the nature of these effects varied with target/movement direction. Some aspects of endpoint variability (i.e., the orientations of movement endpoint distributions), varied in a straightforward way with arm posture, while other aspects (the size and shape of the distributions) were determined by a more complex interaction between initial arm posture and the visual conditions. As discussed below, the results emphasize the interaction of execution noise with feedback and biomechanical factors in constraining patterns of movement variability in 3D space.

### Visual effects

Several previous studies have examined the contributions of planning and execution-related noise to movement variability for both planar (2D) arm movements (Gordon et al., 1994; Vindras et al., 1998; Van Beers et al., 2004) and reaching or other arm-related behaviors in 3D space (Mcintyre et al., 1997, 1998; Carrozzo et al., 1999; van Den Dobbelsteen et al., 2001; Apker et al., 2010; Apker and Buneo, 2012). The results of the 3D reaching studies are most germane to the present one. In the V condition, axes of maximum variability appeared to vary systematically with the direction of the targets relative to the head and/or eyes, rather than with respect to the starting position of the hand. This suggests that variability in this condition was predominantly determined by noise in the initial planning (and/or updating) of hand position in viewer/eye-centered coordinates, rather than noise in hand/arm-centered coordinates, consistent with the conclusions of Mcintyre et al. (1997, 1998). In the NV condition, variability was larger, as expected, and axes of maximum variability were less convergent toward the head. These axes also did not align well with hand movement direction, findings that are also consistent with those of Mcintyre et al. (1997, 1998). This suggests that other factors, e.g., execution related noise, proprioceptive feedback, and final limb impedance, are primary determinants of endpoint variability when hand visual feedback is unavailable.

### Postural effects

In this study, we found that changes in initial arm posture that were orthogonal to planned movement directions were associated with different patterns of movement endpoint variability. Moreover, the nature of these effects differed for different movement directions. Although our primary focus was on potential changes in the orientations of movement endpoint distributions, we also quantified effects of arm posture on the sizes (volumes) and shapes (aspect ratios) of these distributions. With regard to volumes, there was a significant interaction between the visual conditions and initial arm posture, with volumes tending to be larger for the ABD posture in the V condition, and larger for the ADD posture in the NV condition. Similarly, there was a significant interaction between the visual conditions and initial arm posture on aspect ratios, with these ratios tending to be larger for the ADD posture in the V condition and for the ABD posture in the NV condition. Notably, effects on both parameters were only evident for the leftward target.

These interaction effects are best interpreted in the context of previous experiments employing the same experimental setup (Apker et al., 2010; Apker and Buneo, 2012). In those experiments, subjects reached to multiple targets using a single, adducted initial arm posture. Reach endpoint distributions were found to be consistently smaller in volume, more elongated in shape, and more aligned with the depth axis (the axis along which visual planning noise is greatest) when hand visual feedback was provided compared to when feedback was withheld. These findings and others suggested that when feedback was present, noise associated with planning and updating the position of the hand in visual coordinates played a dominant role in determining patterns of endpoint variability. In addition, these results suggested that visual feedback partially mitigates the effects of execution noise, as these effects were more apparent when feedback was withheld.

The results of the present experiment suggest that this interpretation was incomplete. Here we also observed that volumes were smaller in the V condition than in the NV condition, a finding that was consistent between arm postures and across targets. However, for the left target, volumes were larger for the ABD posture than for the ADD posture in the V condition but showed the opposite trends with posture in the NV condition. As a result, the *difference* in volumes between the V and NV conditions was greater for the ADD posture than for the ABD posture (as was the difference in aspect ratios). This implies that visual feedback was less effective at mitigating the effects of execution noise when movements were initiated with the ABD posture. More generally, this observation suggests that the manner in which visual feedback interacts with execution noise depends upon the biomechanical requirements of a given task. This may also explain why the observed interactions were only observed when reaching to one target (i.e., the left one, though a similar trend was observed for the middle target), as the biomechanics involved in reaching to different parts of the workspace with the right arm varied considerably in this experiment.

With regard to the orientations of the endpoint distributions, we found a significant main effect of initial arm posture for the middle target. Here, changing from an ADD to an ABD posture resulted in a counter clockwise rotation of the endpoint distributions in the transverse plane (Figure 5). These distributions also appeared to pitch downward when viewed from the participants’ perspective, but these elevation changes were much more variable across participants than for azimuth. When differences in orientation were analyzed as a function of movement extent (Figure 9A), they were found to be positive soon after movement onset (consistent with a counter clockwise rotation in the transverse plane), reverse sign toward the middle of the movements (implying a clockwise rotation), and reverse sign again at the endpoint. Note that the sign of these differences themselves are not meaningful, as we arbitrarily chose to subtract azimuth values for ADD from ABD (rather than the opposite). However, the sign change toward the middle of the movements *is* likely meaningful, though difficult to interpret without further study. The early orientation differences most likely resulted from posture dependent differences in planning noise. Given that information about target locations was identical between blocks of trials employing different arm postures, it is likely that posture dependent uncertainty regarding initial hand position was the root cause of these early differences. Studies showing that the precision of hand position estimates is anisotropic and posture dependent in both 2D and 3D support this view (Wilson et al., 2010; Klein et al., 2018).

Later differences in orientation likely reflect the effects of posture dependent execution noise. Harris and Wolpert (1998) have proposed that neural control signals are corrupted by noise whose variance scales with control signal magnitude, a phenomenon referred to as “signal-dependent” execution noise (Harris and Wolpert, 1998). In this scenario, larger control signals, e.g., greater degrees of muscle activation, are expected to produce faster movements but also greater levels of noise, resulting in larger variance in endpoint position. Interestingly, in the present experiments, movements to the middle target differed in peak movement velocity when initiated with different arm postures (Supplementary Table 2), but this velocity difference was not associated with concomitant differences in overall variability, as judged by ellipsoid volumes (Table 1). This suggests that posture dependent differences in ellipsoid orientation observed for movements to the middle target did not result solely from signal-dependent noise but likely reflect the influence of both signal-dependent and signal-*independent* processes (“constant” and/or “temporal” noise), as suggested by previous studies (Van Beers et al., 2004).

Biomechanical factors, specifically the limb’s mechanical impedance at the endpoint, may also have contributed to some aspects of the posture dependent variability observed here. In the context of limb motor control, mechanical impedance refers to the limb’s effective mass, inertia, stiffness, and damping, which is believed to be an important factor influencing the performance motor tasks (Mizrahi, 2015). Limb impedance has previously been implicated as a factor influencing behavior (including variability) during planar arm movements (Scheidt and Ghez, 2007; Lametti and Ostry, 2010) and is arm position/configuration dependent in both 2D and 3D space (Mussa-Ivaldi et al., 1985; Artemiadis et al., 2010). Our analyses, which showed that differences in overall orientation and azimuth were correlated with differences in mean endpoint position, is consistent with the idea that position dependent differences in final limb impedance contributed to differences in variability. A stronger relative contribution of final limb impedance to variability in the NV condition could also help explain our observation that the orientations of axes of maximum variability did not align well with the eyes/head or planned movement directions in that condition.

#### Direction dependent effects

Based in part on the results of previous simulation studies (Shi and Buneo, 2012), we expected that endpoint distributions associated with different movement directions would show similar dependencies on arm posture, though the magnitude and direction of these expected effects were not predictable *a priori* given the nature of the change posture. Instead, we found that effects of arm posture were not uniform across directions, e.g., endpoint distributions only showed a consistent pattern of rotation with changes in arm posture for the middle target. What could explain this discrepancy? One possible factor concerns the nature of our simulations themselves. That is, these simulations were entirely feedforward in nature, i.e., they did not incorporate feedback control [neither for the trajectory nor for final position (Scheidt and Ghez, 2007)]. As a result, the analyses of our simulation data focused on variability in initial movement directions, rather than variability in movement endpoints. In contrast, movements in the present study were closed loop with respect to proprioceptive feedback (in both conditions) and visual feedback in the V condition. Thus, it is conceivable that online feedback processes interact with execution related ones in such a way that effects of arm posture on movement endpoint variability are inconsistent across directions, at least for the changes in arm posture employed here.

## Data availability statement

The raw data supporting the conclusions of this article will be made available by the authors, without undue reservation.

## Ethics statement

The studies involving human participants were reviewed and approved by the Arizona State University Institutional Review Board (IRB). The patients/participants provided their written informed consent to participate in this study.

## Author contributions

PP collected the data, analyzed the data, interpreted results of the experiments, drafted and edited the manuscript. QR and KL conceived and designed the experiments, interpreted results of the experiments, and edited the manuscript. MF collected the data, interpreted results of the experiments, and edited the manuscript. CB conceived and designed the experiments, collected the data, analyzed data, interpreted results of the experiments, prepared figures, and drafted the manuscript. All authors contributed to the article and approved the submitted version.
